# The Actions of Lyophilized Apple Peel on the Electrical Activity and Organization of the Ventricular Syncytium of the Hearts of Diabetic Rats

**DOI:** 10.1155/2016/8178936

**Published:** 2015-12-29

**Authors:** Elideth Martínez-Ladrón de Guevara, Nury Pérez-Hernández, Miguel Ángel Villalobos-López, David Guillermo Pérez-Ishiwara, Juan Santiago Salas-Benito, Alejandro Martínez Martínez, Vicente Hernández-García

**Affiliations:** ^1^Institute of Biomedical Sciences, Autonomous University of Ciudad Juárez, 32310 Ciudad Juárez, CHIH, Mexico; ^2^National School of Medicine and Homeopathy, National Polytechnic Institute, 07320 Mexico City, DF, Mexico; ^3^Centre for Research in Applied Biotechnology, National Polytechnic Institute, 90700 Tepetitla, TLAX, Mexico

## Abstract

This study was designed to examine the effects of lyophilized red delicious apple peel (RDP) on the action potentials (APs) and the input resistance-threshold current relationship. The experiments were performed on isolated papillary heart muscles from healthy male rats, healthy male rats treated with RDP, diabetic male rats, and diabetic male rats treated with RDP. The preparation was superfused with oxygenated Tyrode's solution at 37°C. The stimulation and the recording of the APs, the input resistance, and the threshold current were made using conventional electrophysiological methods. The RDP presented no significant effect in normal rats. Equivalent doses in diabetic rats reduced the APD and ARP. The relationship between input resistance and threshold current established an inverse correlation. The results indicate the following: (1) The functional structure of the cardiac ventricular syncytium in healthy rats is heterogeneous, in terms of input resistance and threshold current. Diabetes further accentuates the heterogeneity. (2) As a consequence, conduction block occurs and increases the possibility of reentrant arrhythmias. (3) These modifications in the ventricular syncytium, coupled with the increase in the ARP, are the adequate substrate so that, with diabetes, the heart becomes more arrhythmogenic. (4) RDP decreases the APD, the ARP, and most syncytium irregularity caused by diabetes.

## 1. Introduction

The cultivation of the apple (*Malus domestica*) for human consumption dates back centuries, and apples are now estimated to be the third most commonly consumed fruit, after bananas and citrus [1]. Epidemiological studies have related the ingestion of one apple or more a day to the prevention of pulmonary and colon cancers [2], cardiovascular disease, type 2 diabetes, pulmonary disorders, and Alzheimer's disease [3]. Some apple components have even been found to have beneficial effects with regard to cognitive loss with ageing, osteoporosis, gastrointestinal protection, and the maintenance of body weight [4]. The protective effects are attributed to phytochemical compounds such as triterpenes and polyphenols due to their antioxidant properties. Furthermore, the apple contains large amounts of free polyphenols [5]. However, the composition of phytochemicals depends on factors including the variety, cultivation conditions, harvesting, soil, and climate, in addition to the type of fruit tissue that is considered (peel, pulp, and seed). The peel contains the greatest amount of polyphenols because it is the main physical, chemical, and biological protection of the fruit from the external environment [1].

The relationship between oxidative stress with diabetes and its micro- and macrovascular complications has been known for years, but the mechanisms involved have only relatively recently begun to be understood. Interestingly, the redox state and diabetic cardiomyopathy that involves both mechanical and electrical cardiac dysfunction [6] are closely related [7] and are responsible for an increased vulnerability to developing heart arrhythmias and sudden death. Nonetheless, to date, there has been only one study, which indicates that red delicious apple peel (RDP) has direct actions on the heart's electrical activity with diabetic cardiomyopathy [8]. Within this context, the objectives of this study are (1) to evaluate and compare the action of the 50 mg/kg dose of RDP on the duration of cardiac action potentials (APs) at 30, 50, and 90% in normal and diabetic rats and (2) to analyse the functional organization of the ventricular syncytium through the input resistance versus threshold current curve of the hearts of control rats, control rats with RDP, diabetic rats, and diabetic rats with RDP administered daily for 90 days. The results obtained indicate that (1) RDP induced a significant decrease in action potential duration (APD) and absolute refractory period (ARP); (2) the cardiac syncytium in healthy animals and in those with diabetes is heterogeneous, in terms of input resistance and intracellular thresholds. However, in animals with diabetes, this irregularity is more accentuated; and (3) RDP administered orally to diabetic rats attenuates the irregularities of the relationship between input resistance and threshold current.

## 2. Methods

### 2.1. Apple Peel

The production of apple varieties in Mexico is mainly concentrated on the Golden Delicious and Red Delicious varieties [9]. Only red apple varieties contain anthocyanins; these flavonoids are responsible for the red and blue tones occurring in various fruits, such as grapes, berries, and figs. For this reason we decided to use the Red Delicious variety, which also has the highest concentration of free polyphenols [10–12].

Red Delicious apples, harvested in 2011, were purchased in the market of Ciudad Juarez, CHIH. To obtain the apple peels, a manual peeler was employed once the fruit was washed. The peels were immediately stored at −80°C. The lyophilization was performed using a 6-litre Labconco FreeZone lyophilizer. Once the peels were lyophilized, they were pulverized to obtain a fine powder that was vacuum-packed in plastic bags, shielded in black plastic bags, and stored at −20°C until being utilized. Thus, the biological activity of the polyphenols was preserved.

The proximate analysis of the RDP was performed according to the official methodology outlined by the* Association of Official Analytical Chemists* (AOAC) [13]. The total nitrogen was determined by the Kjeldahl technique, and the crude protein was calculated by multiplying the total nitrogen by 6.25. The crude fat was quantified by the Soxhlet technique. The carbohydrates were quantified by means of the difference of the other components. The fibre was quantified after their acid and alkaline digestion.

Phenol extraction was performed using 20 g of RDP dissolved in 125 mL of 80% methanol. The total phenolic content was determined with the modified Folin-Ciocalteu colorimetric method [14]. The measurement was compared to a standard curve of gallic acid, and the results were expressed in units of mg of gallic acid equivalent (GAE) per 100 g dry weight.

The determination and quantification of the isomers of chlorogenic acid and epicatechin were performed in the laboratories of the* Silliker* Company at* Mérieux NutriSciences* in Grand Prairie, TX, USA.

### 2.2. Animal Model

The male Wistar rats that were used were acquired from* Rismart* and* Research Global Solutions* in Mexico City. The rats were maintained at a constant temperature of 25°C with a LD 12 : 12 cycle. Both food and water were always offered* ad libitum*. The treatment period was equivalent to 90 days, and 36 rats were utilized. They were divided into four groups: control (*n* = 10), control with RDP treatment (*n* = 4), type 1 diabetes (*n* = 11), and type 1 diabetes with RDP treatment (*n* = 11).

#### 2.2.1. Groups with Apple Peel

The groups treated with RDP received a daily dose of 150 mg/kg. The concentrate was dissolved in distilled water in small volumes and administered orally with a tuberculin syringe. The dose was equated to the daily consumption of the peels of three apples/day ingested by an adult man weighing 70 kg.

#### 2.2.2. Induction of Diabetes Mellitus

The induction of diabetes mellitus type 1 was accomplished using a single intraperitoneal administration of 45 mg/kg of streptozotocin (STZ,* Sigma Chemical Company*) dissolved in a citrate buffer at pH 4.8. To decrease the mortality due to the hypoglycaemia generated by the STZ, 60 mM of sugar water was provided* ad libitum* for one week. The glycaemia of the blood was determined at eight days after STZ injection using a commercial blood glucose metre (*OneTouch Ultra 2*). For this study, only those rats with glycaemia values above 150 mg/dL were used. All experiments were performed according to the guidelines established by the Ethics Committee for Experimental Animals of the Autonomous University of Ciudad Juárez.

#### 2.2.3. Electrophysiological Experiments

Prior to experimentation, the rats were anesthetized with sodium pentobarbital at 50 mg/kg and 0.2 units of heparin/mL. Once an animal was anesthetized, its abdomen was opened; from this area, cardiac puncture was performed, and a 4 mL blood sample was obtained. Then, proceeding to the isolation of the heart, it was placed in an isolated tissue chamber and continuously perfused with oxygenated Tyrode's solution at 37°C. Under these conditions and by dissection of the left ventricle, the left ventricular papillary muscles were isolated. The preparation thus obtained was stimulated through external bipolar silver electrodes coated with insulating material, except for the tip, at a basic cycle of 500 milliseconds. The stimuli were rectangular pulses of one millisecond in duration and an intensity 1.5 times the threshold, obtained from a Digitimer model D4030 pulse generator and passed through a Digitimer model DS2 stimulus isolation unit. When the propagation of premature responses (extrasystoles) was explored, the preparation was stimulated regularly at a basic cycle of 500 milliseconds and, after applying eight basic stimuli, a test stimulus was introduced through the same pair of stimulation electrodes at different time intervals. The intracellular action potentials were obtained using glass microelectrodes filled with 3 M KCl solution and with a tip resistance of between 10 and 20 megaohms. The signal obtained by the microelectrodes was passed through high input impedance amplifiers, WPI model 750, and an HV Electrometer model 400E. The recording of the APs was performed on the screen of a Tektronix model TDS3034B oscilloscope. The records were stored on a Dell model GX520 computer for further quantitative analysis.

#### 2.2.4. Biochemical Analyses

The quantifications of glucose and glycosylated haemoglobin (HbA_1c_) were determined at 90 days by spectrometry in the Servalab Clinical Laboratory (Puebla, Mexico). To determine the glucose concentration, a Glucose PAP kit (ELITech Clinical Systems) was used in a Vitros DT60 Chemistry System, whereas the HbA_1c_ was performed with a LabonaCheck A_1c_ kit in an HbA_1c_ Analyser. The evaluation of the plasma insulin levels was performed by chemiluminescence in the Italo Gaya Laboratory (Puebla, Mexico).

### 2.3. Statistical Analysis

The data were analysed with GraphPad Prism version 4.00 for Windows (GraphPad Software, La Jolla, California, USA). The numerical results are expressed as mean values ± SEM (standard error of the mean). The differences observed between the control and the treated groups were assessed using unpaired two-tailed Student's* t*-test. Values were considered statistically significant at *p* < 0.05.

## 3. Results

### 3.1. Analysis of the Apple Peel

Table 1 shows the principal components and their values in % of RDP. The polyphenol content was 1,100 *μ*g GAE/100 g dry weight. Specifically, the isomers of chlorogenic acid and epicatechin were present at a concentration of 0.1148 and 2.35 mg/g dry weight, respectively. The determination of the total polyphenols by means of the Folin-Ciocalteu method depends on the extraction of the polyphenols and the units used to report them; thus, it is difficult to compare the results. However, the levels of chlorogenic acid and epicatechin in the peels are in line with those published by other authors [2]. Both chlorogenic acid and epicatechin are the most abundant polyphenols in the apple [4].

### 3.2. Criteria for Considering Diabetogenesis in Wistar Rats

Table 2 presents variables relating to the body weight and biochemical blood parameters of the rats 90 days after diabetes mellitus was induced.

### 3.3. Action Potentials

#### 3.3.1. Characteristics of the Action Potentials (APs) of the Hearts of the Control Rats and the Diabetic Rats

**Figure 1:**
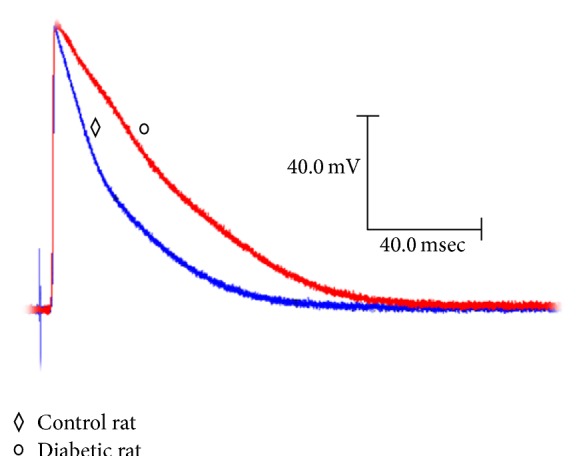
Changes in the action potential with diabetes. Representative action potentials obtained from a cell of the papillary muscle of the left ventricle of the heart of control rats and diabetic rats at a basic cycle of 500 milliseconds.

Figure 1 shows the overlapping mean APs, recorded in the isolated papillary muscles of hearts from the control rats (blue diamond) and the diabetic rats (red circle), evoked by a basic 500-millisecond cycle.

**Figure 2:**
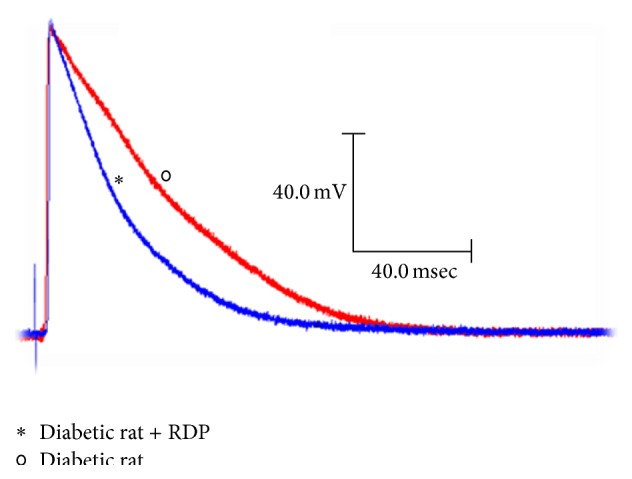
Action of the apple peel on the action potentials of diabetic rats. Typical transmembrane potentials obtained in the papillary muscles of the heart of diabetic rats and diabetic rats with apple peel at a basic cycle of 500 milliseconds.

Figure 2 shows the result of averaging the APs of the diabetic rats and the diabetic rats treated for 90 days with 150 mg/kg of RDP.

**Figure 3:**
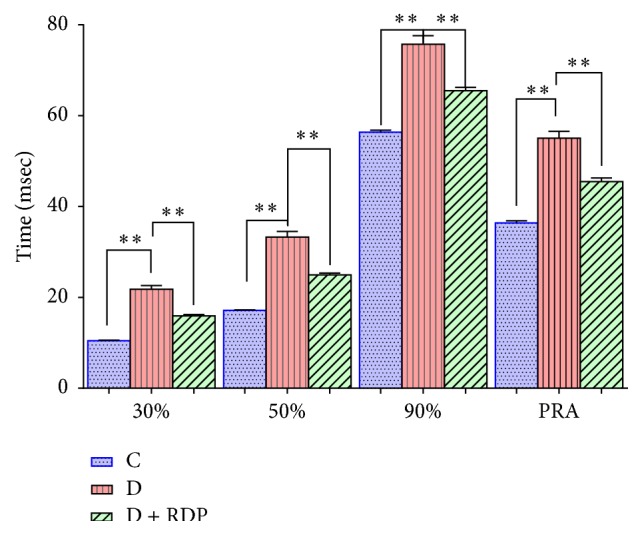
Mean values ± SEM of the quantified parameters of the action potentials recorded in the papillary muscles of the hearts of control rats (*n* = 225), diabetic rats (*n* = 110), and diabetic rats receiving apple peel for 90 days (*n* = 169) at a basic cycle of 500 milliseconds. The symbols (*∗∗*) indicate that the differences between the different groups are significant (*p* < 0.05).

Figure 3 summarizes the APD at 30, 50, and 90% and the ARP of the control rats, the diabetic rats, and the diabetic rats treated with RDP.

### 3.4. Modifications Occurring in the Ventricular Syncytium Muscle with Diabetes

#### 3.4.1. Determination of the Input Resistance and Intracellular Thresholds in the Papillary Muscles of Control Rats and Diabetic Rats Using the Method [14]

Concisely, (1) the papillary heart muscles of the rat present a syncytial geometric organization and (2) to evaluate the possible potential changes of this syncytial organization, it is necessary to quantify the parameters of the input resistance and the threshold current (necessary to evoke a propagated AP).

To quantify the input resistance and the threshold current, two microelectrodes penetrating the same cell were used. The microelectrodes were cemented or glued, aligned under a microscope, and separated between their tips at a distance of approximately 10 *μ*m. The microelectrodes were mounted in a double micromanipulator (Narishige MD-4) with independent vertical movement. With this system, it was possible to impale the two microelectrodes in the same cell or in two adjacent cells connected by their* nexus*. Under these strict conditions, depolarizing or hyperpolarizing current pulses were injected through one microelectrode, and, with the other, the changes in the transmembrane potential were recorded.

**Figure 4:**
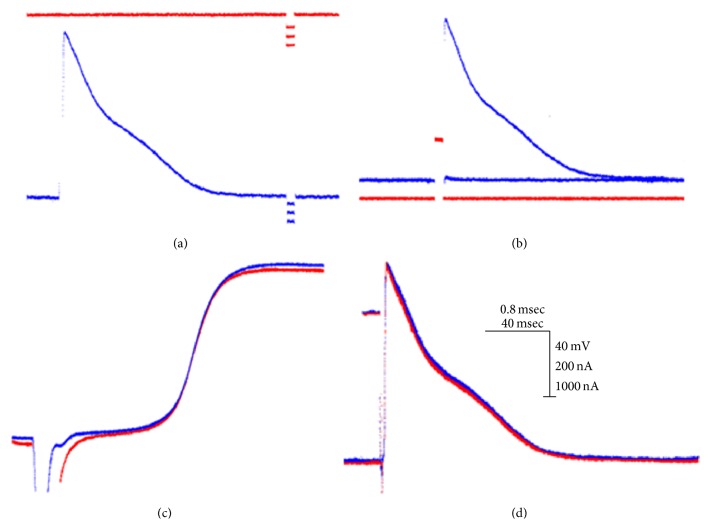
Procedure for determining the input resistance. Steps followed to quantify the input resistance and the intracellular thresholds in the papillary muscles of the hearts of the control rats: (a) injection of the pulse current in late diastole to evaluate the input resistance; (b) determination of the intracellular threshold; (c) simultaneity and shape of the depolarization phase of the action potentials; (d) zero potential of both records obtained from the same cell.

Figure 4 shows the steps that were followed to quantify the input resistance and the intracellular thresholds in the papillary muscles of the control rats. The top trace in Figure 4(a) shows the injection of three constant current hyperpolarizing pulses, in late diastole, with a duration of 7.0 milliseconds and intensities of 35, 68, and 100 nanoamperes, and their corresponding variations in the membrane potential of 5, 10, and 16 millivolts (lower trace). By plotting the change in membrane potential with respect to the injected current, a straight line is obtained, with a slope that is the value of the input resistance. In Figure 4(b), the current polarity is inverted, and a pulse of depolarizing current is injected. The pulse duration is 7.0 milliseconds, and its intensity is gradually increased until it can evoke an action potential. In this case, the intracellular threshold is 805.5 nanoamperes.  Figure 4(c) shows the simultaneity and the same configuration of the depolarization phase of the action potentials recorded in the same cell by both of the microelectrodes. Finally, in Figure 4(d), the zero potential of both action potentials recorded in the same cell is obtained. Therefore, it is important to note that the value of the input resistance of the cell will be valid provided that the requirements shown in Figures 4(c) and 4(d) are met.

**Figure 5:**
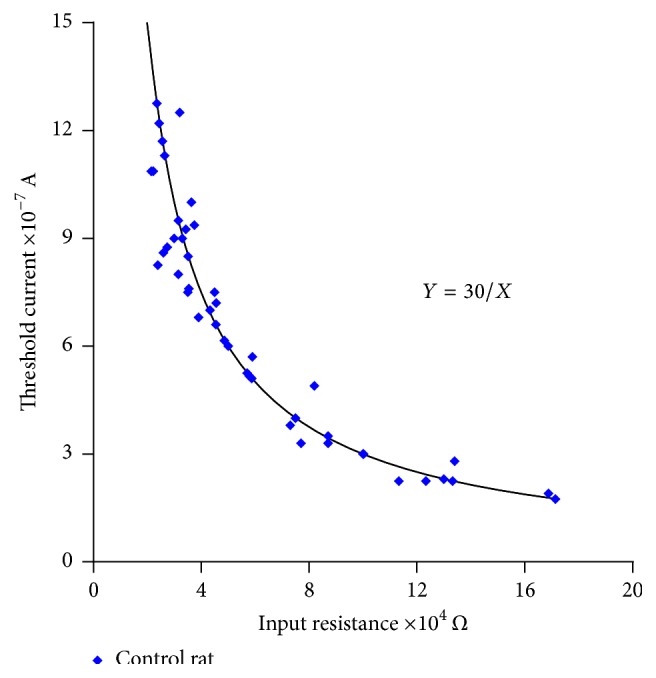
Fit relationship of the experimental input resistance-current threshold values (blue) with respect to the theoretical curve (black). Values obtained in the cells of the papillary muscle of the heart of the control rats. The continuous curve corresponds to an equilateral hyperbola defined by the equation *Y* = *K*/*X*, where *K* = 30 mV. For each input resistance value, there is a corresponding threshold current value adequately conforming to the theoretical curve, which is established by the statistical analysis of *R* ^2^ = 0.92 and *p* < 0.05.

Using this procedure, sufficient data on the input resistance and threshold current were obtained in the control rats to show that their product (ohms × amperes = volts) equals a mean value of 30 mV. In other words, the experimental results are fitted to the theoretical curve of an equilateral hyperbola in the form *Y* = *K*/*X*, where *K* is a constant with a value equalling 30 mV; and the *XY* product (resistance × current) is the drop in potential that corresponds to the value of the constant, *K*, with units in volts. These experimental relationships are shown in Figure 5, with a function, *F*, and *p* < 0.05. In this graph, the experimental values of the input resistance ranged between 24 and 171.4 KΩ, and the minimum current threshold necessary to initiate propagated responses ranged from 175 to 1,220 nanoamperes.

Because the preparation of the papillary muscle of the rat heart maintains its force of contraction, the duration of the impalement of the microelectrodes is brief. Determining the threshold is achieved by small amplitude steps of increasing current until initiating an AP; consequently, our threshold values lacked absolute precision. Therefore, if the input resistance and threshold current are adequately related, with the equation of an equilateral hyperbola with constant value, *K* = 30 mV, then we can quantify the intracellular thresholds more precisely and explore more of the cells in the papillary muscles, impaling two independent microelectrodes at different sites in the preparation. Thus, we can inject the current in smaller steps through one electrode up to the threshold and record the propagated AP through the other. Under these conditions, the input resistance values can be calculated with the equation of the equilateral hyperbola: *Y* = *K*/*X*.

#### 3.4.2. Relationship between Input Resistance and Threshold Current in the Hearts of the Control Rats and the Control Rats with Treatment

To make the electrode impalement technique more efficient, obtain a greater amount of experimental data, and have more precise current threshold values, the microelectrode recording the APs remains fixed somewhere in the biological preparation, and the other microelectrode is impaled into many other sites of the papillary muscle for the determination of their thresholds. These data are now used to calculate their corresponding input resistance.

**Figure 6:**
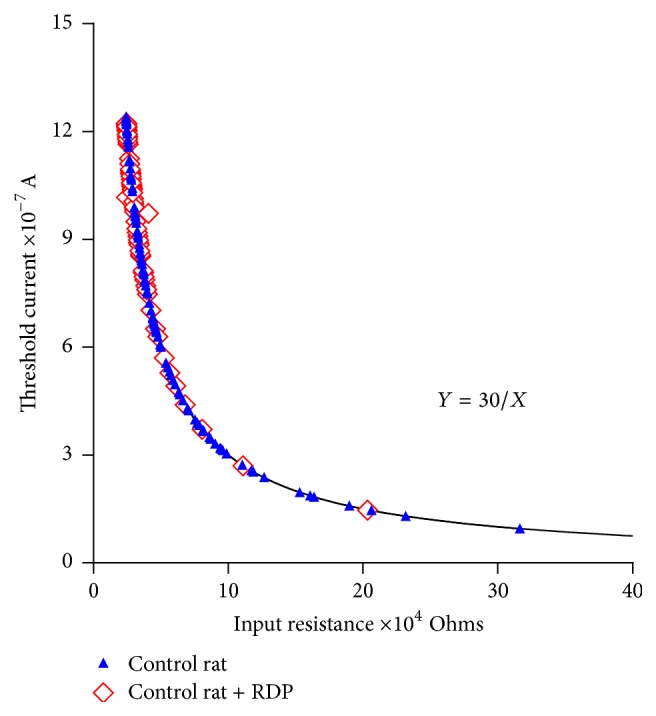
Input resistance-current threshold relationship in control rats and control rats with RDP treatment (150 mg/kg). The values of the input resistance were calculated using the equation of the equilateral hyperbola, *Y* = *K*/*X*, where *Y* is the threshold current quantified experimentally and *K* = 30 mV is the constant value. Filled symbols: control rats; unfilled symbols: control rats + RDP.

Figure 6 shows the relationships between the papillary muscles of the control rats and the papillary muscles of the control rats treated with 150 mg/kg of RDP over a 90-day period. The values for the input resistance found in the control rats are between 24 and 316.5 KΩ, and their corresponding threshold currents range from 94 to 1,232 nanoamperes. Similar input resistance and threshold current values were observed in both groups.

#### 3.4.3. Relationships between Input Resistances and Threshold Currents in the Control Rats and the Diabetic Rats

**Figure 7:**
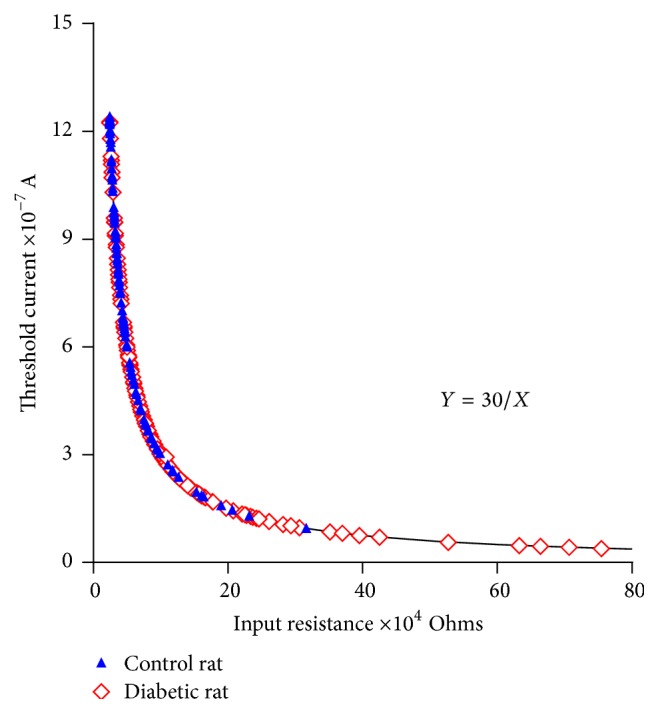
Input resistance versus threshold current relationship in the control rats and diabetic rats. The relationship obtained in the papillary muscle of the heart of the control rats and the rats with diabetes. The continuous line corresponds to the theoretical equilateral hyperbola, *Y* = 30/*X*. Filled symbols: control rats; unfilled symbols: diabetic rats.

Figure 7 shows the changes between the input resistance and threshold current values due to the diabetogenic effects. Wide variations in input resistances, ranging from 24 to 814.1 KΩ, in threshold currents, ranging from 36 to 1,232 nanoamperes, and the resting membrane potential of −77.50 ± 0.3554 mV (*n* = 109) were found in the papillary muscles of the diabetic rats, while in the control rats the variations in input resistance range from 24 to 316.5 KΩ and corresponding threshold current ranges from 1,232 to 94 nanoamps with a resting membrane potential of −76.62 ± 0.5664 mV (*n* = 252). Observe the displacement towards higher input resistance values and low thresholds in the hearts of the diabetic rats.

#### 3.4.4. The Input Resistance-Threshold Current Relationship in the Diabetic Rats and the Diabetic Rats with RDP Treatment

**Figure 8:**
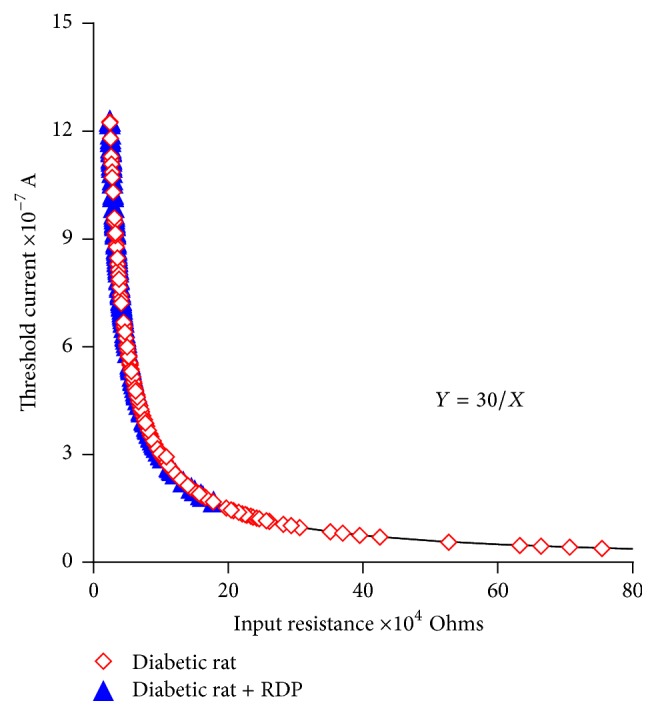
Changes in the input resistance-current threshold relationships in diabetic rats and diabetic rats treated with RDP; 150 mg/kg for 90 days. Filled symbols: diabetic rats; unfilled symbols: diabetic + RDP.

Figure 8 shows the effects of RDP on the input resistance-threshold current values obtained in the papillary muscles of the diabetic rats and the diabetic rats treated with RDP. The results clearly indicate a reduction in diabetogenic effects due to the administration of the RDP, based on the values of the input resistance and threshold current.

#### 3.4.5. Propagation of Premature Responses and Reentrant Activity in the Diabetic Rats

The modifications in the input resistance and the intracellular threshold found in the papillary muscles of the rats with diabetes can cause the propagation of APs that may become critical; under these conditions, reentrant bioelectrical activity may arise which initiates ventricular fibrillations and sudden death.

**Figure 9:**
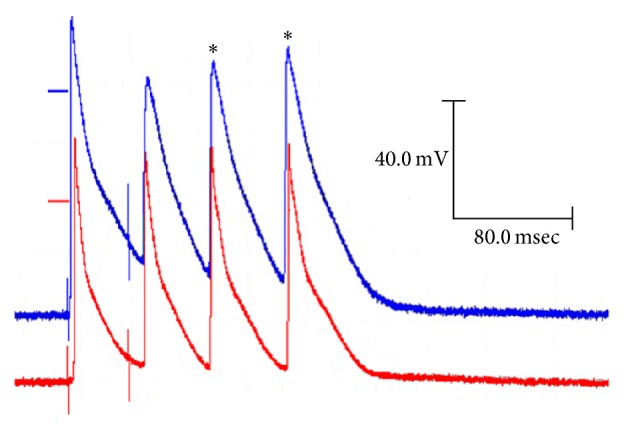
Reentrant activity initiated by the application of an early extrasystole. The traces shown correspond to action potentials recorded in the papillary muscles of diabetic rats, in the area proximal (upper trace) and the area distal (lower trace) to the stimulation site, at a basic cycle of 500 milliseconds. Observe that, after the implementation of the extrasystole, two responses (asterisks) not evoked by stimulation appear, corresponding to a type of reentrant activity.

Figure 9 shows the response evoked by the application of an early extrasystole in the papillary muscle of diabetic rats. The upper trace corresponds to the APs recorded in the area proximal to the site of the external stimulation electrodes; the lower trace corresponds to the APs recorded in a site distal to the stimulation electrodes. The first response corresponds to the last of a series of eight APs evoked by basic stimuli. The second response corresponds to the activity generated by a test stimulus applied 40 milliseconds after the last basic stimulus. It is observed that the AP evoked by the test pulse is followed by two APs (asterisks) that were not initiated by stimulation, which demonstrates the hypersensitivity of the ventricular syncytium of diabetic rats to generate reentrant action potential activity.

**Figure 10:**
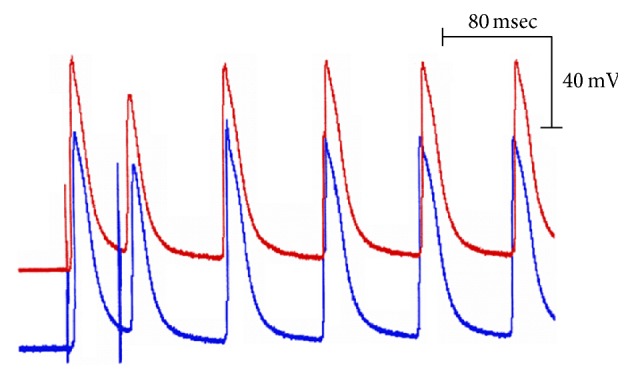
Change of direction in the propagation of the wavefront. Observe that the first three responses (basic, test, and first reentrant) are propagated from the stimulation site towards the rest of the preparation, whereas the last three following reentrant responses propagate in the opposite direction.

Another example of reentrant activity is shown in Figure 10. This result provides additional evidence that when a test pulse is applied early, the evoked response is followed by multiple reentrant activity. In this case, the last two reentrant action potentials are conducted in a retrograde direction.

## 4. Discussion

This study was designed with the purpose of obtaining solid experimental evidence of the effects of RDP on diabetic cardiomyopathy. A well-established model of diabetes induced by streptozotocin in the rat was used for this purpose [15, 16]. The effects of RDP were quantified with the electrophysiological parameters of the papillary muscle of the hearts of male Wistar rats. The electrophysiological properties studied included (1) the duration of the ARP and APs at 30, 50, and 90% of their repolarization and (2) the organization of the ventricular syncytium muscle in terms of the input resistance-current threshold relationship in control rats, control rats treated with RDP, diabetic rats, and diabetic rats treated with RDP.

It is important and necessary to clarify that we are unable to provide an adequate explanation of or the mechanisms that are involved in the actions and effects of the RDP for the following reasons: (1) This is the first study in which the action of RDP is assessed. (2) All of the biologically active components of RDP are not known. (3) Their concentrations and pharmacokinetic and pharmacodynamic properties prevent us from formulating an explanation. Considering the foregoing and with the aforementioned reservations, we interpret the actions of RDP on the hearts of diabetic rats.

### 4.1. Action Potentials

The results obtained clearly show that the duration of the action potentials increases in the papillary heart muscles of the diabetic rats compared to the control rats (Figures 1 and 3) [16–20]. Additionally, the increase in the duration of the action potential is more pronounced at 30% than at 90%. There are several ionic currents that intervene spatially and temporally in the repolarization of the action potential in the ventricular myocardium of the rat. The early phase of ventricular repolarization is performed by the activation of two potassium currents (*I*_K_^+^), the transient outward current (*I*_to_) and the delayed rectifier current (*I*_K_) [21], whereas the late phase of repolarization is due to the activation of the Na^+^/Ca^2+^ exchanger current, which is responsible for the final elongation of the repolarization of the action potential [22]. Furthermore, the increase observed in the early phase of the repolarization of the action potentials in the diabetic rats is due to the decrease in the potassium currents, *I*_to_ and *I*_K_ [16, 18, 20], whereas the increase in APD_90%_ is attributed to the increase in the Na^+^/Ca^2+^ exchanger current_,_ causing an overload of Ca^2+^ in the ventricular myocytes [23–25].

Figures 2 and 3 show that the supplementation of apple peels to the diabetic rats for 90 days after having induced diabetes significantly decreased the duration of the action potentials and the ARP. Results similar to those shown in this study have been obtained by other authors [26]. It has been reported that the polyphenols contained in apple extract decrease the duration of the action potential in the ventricular myocytes of the mouse heart with dilated cardiomyopathy. Such a decrease in the APD is the result of an increased K^+^ current (*I*_K1_), induced by the polyphenols extract. The results obtained in the papillary muscles of diabetic rats with treatment can be explained if we assume that the RDP has the same types of polyphenols and is at concentrations similar to those found in the apple extracts tested [25]. Consequently, the RDP could cause an increase in the transient outward K^+^ current (*I*_k_to__), given that it is the principal K^+^ current affected in the ventricular myocytes of the hearts of diabetic rats [16, 20]. Indeed, it is necessary to measure this current in the ventricular myocytes of the diabetic rats to provide a more sustainable affirmation.

### 4.2. Organization of the Ventricular Syncytium of the Rat Heart

Our study was conducted in the isolated papillary muscles of the left ventricle of the rat heart. Before considering the interpretation of the results, in which the organization of the ventricular functional syncytium was analysed, we must consider the following factors:

1. The first work in which the organization of the ventricular functional syncytium was analysed [27] was developed in the right anterior papillary muscle of the dog heart. This preparation has been used extensively in studies of heart electrophysiology. The morphologies of the intracellular action potentials revealed the existence of three functionally distinct areas in the right anterior papillary muscle of the dog heart [28]. The proximal and middle thirds are composed of muscle tissue and specialized conduction tissue. The distal third contains only ventricular muscle. The same authors designated the end portion of the conduction tissue as terminal Purkinje fibres. The terminal Purkinje fibres establish low-resistance electrical contact with the ventricular muscle fibres and give rise to the Purkinje-muscle junctions. In this manner, the preparation of the papillary muscle of the dog provides a syncytium composed of different cellular elements that can be easily identified.
2. Nonetheless, the rat is an experimental model widely used in cardiac electrophysiology studies. To date, there is no interest in performing the classification made in the anterior papillary muscle of the dog in the papillary muscle of the left ventricle of the rat [28]. With that condition, it is assumed that there is a similar functional structure in their proximal and middle thirds (composed of specialized conduction fibres and ordinary ventricular muscle cells). This asseveration is supported by the similarity of the input resistance values evaluated in the proximal and middle thirds (high resistance values and low thresholds). Low resistance values and high thresholds are found in the distal third, which indicates that the distal third may possibly be composed exclusively of muscle tissue. Thus, the homology is considered applicable in all mammals.

#### 4.2.1. The Input Resistance-Threshold Current Relationship in the Papillary Muscles of the Control Rats

The knowledge of the structural organization of the ventricular syncytium is made possible through the study of its active and passive functional properties. The foregoing involves first determining the characteristics of the generation and propagation of its APs. In making these determinations, understanding its behaviour as a functional syncytium is favoured, in addition to the importance that it represents for the structural geometry of the organization of the cardiac ventricular syncytium. It also helps to explain, under normal conditions, the proper propagation of the APs, even when there is a low margin of safety for the propagation [29].

It was observed that the experimental data on the input resistance-threshold current obtained in the cardiac syncytial system of the rat fit an equilateral hyperbola (Figures 5, 6, 7, and 8) and that the values of the input resistance and intracellular thresholds change, with relatively broad ranges.

These results show the following characteristics of the mammalian ventricular functional syncytium [27]:

(1) The input resistance and threshold current are inversely related.
(2) The functional organization of the ventricular syncytium in the left papillary muscle of the rat heart is irregular in terms of the values of the input resistance and the current threshold for initiating an action potential. However, they maintain the same constant value; *K* = 30 mV.
(3) In terms of the threshold current, the cellular excitability is different in each of the explored sites of the papillary muscle preparation.

Therefore, in relatively small areas, the extension of the abundance of low resistance junctions can be an important parameter that can determine and explain the hyperbolic nature of the relationship between the input resistance and the current threshold [27]. With this experimental evidence, it is possible to explain the results obtained. We conclude that the papillary heart muscles from the control rats constitute an irregular syncytium and that the principal cause for this lack of homogeneity is the possible nonuniformity of the spatial distribution of the* nexus* (Figures 5 and 6). Similarly, the experimental data indicate that the smaller the value of the input resistance, the higher the threshold current. The only way we can explain this fact is by assuming that the density of the* nexus* varies from one small area to the next. The small areas that we refer to would be the amount of cells needed to permit the formation of the wavefront [30], and, under these circumstances, every initiation of a wavefront will have a different threshold and, consequently, will present a different* nexus* density. The results shown in Figures 5 and 6 indicate the lack of homogeneity in the ventricular syncytium of the hearts of the control rats.

#### 4.2.2. The Input Resistance-Threshold Current Relationship in the Papillary Muscles of the Diabetic Rats

The irregularity of the ventricular syncytium of the hearts of healthy rats does not imply ventricular electrophysiological abnormalities. However, under pathological conditions such as diabetes (Figure 7), the heterogeneity of the cardiac syncytium is accentuated in these new conditions and increases the probability of the development of lethal arrhythmias. The greater heterogeneity found in the heart of the diabetic rats includes areas of tissue in the papillary muscle with higher input resistance values and lower intracellular thresholds, which implies the existence of areas of tissue with less* nexus* density in the papillary muscle. This phenomenon, observed with high input resistance values and low threshold current values, is firmly supported by studies in diabetic rats in which the decreases in the expression of connexin 43 were found, in addition to the redistribution of their* nexus* [31, 32].

#### 4.2.3. Reentrant Activity in the Ventricular Syncytium of the Diabetic Rats

The accentuated changes in the values of the input resistance and the intracellular thresholds found in the papillary muscles of the diabetic rats become critical to the propagation of the APs. Under these conditions, a wavefront originating from a low-resistance area (high threshold) to high-resistance area (low threshold) propagates easily. However, the opposite case, in which an area of high resistance (low threshold) comes into contact with an area of low resistance (high threshold), faces greater difficulty in propagation because the depolarizing current provided by the wavefront is insufficient for reaching the threshold. Consequently, blocking of the propagation occurs at the site of low resistance, and reentrant activity is facilitated [33]. Figures 9 and 10 clearly show that this phenomenon occurs.

With regard to the results obtained in the papillary muscles of the diabetic rats, it follows that the phenomena exhibited in the propagation of premature responses are the result of discontinuous propagation. Under these conditions, two factors that can cause conduction block in the ventricular syncytium are added: (1) the small efficacy of the premature action potentials, as physiological stimulus, and (2) the irregularity in the input resistance and the intracellular threshold for initiating propagated action potentials (cellular excitability). It is well known that the propagation of the AP in areas of tissue whose excitability is found to be reduced (low input resistance) is performed through electronic potentials [30] and that the magnitude and temporal development of such potentials depend on the organizational geometry of the syncytium.

This study presents two important findings: (1) simultaneous modifications in the input resistance-intracellular threshold in the hearts of diabetic rats and (2) the alterations in the propagation of premature responses. These phenomena allow us to provide an adequate explanation of the arrhythmogenic phenomenon shown by the heart in pathological situations such as diabetes; furthermore, they produce a partial understanding with respect to the high vulnerability of the heart in presenting ventricular fibrillation and sudden death.

#### 4.2.4. Action of the RDP on the Input Resistance-Threshold Current Relationship in the Papillary Muscles of the Hearts of the Control Rats and the Diabetic Rats

Under normal conditions, the daily ingestion of at least one apple is sufficient to decrease the incidence of cardiovascular disease and/or diabetic cardiomyopathy. The dose of RDP provided to the healthy rats for 90 days maintained the heterogeneity of the ventricular syncytium observed in the papillary muscles of the healthy rats (Figure 6). However, the oral supplementation of RDP to the diabetic rats attenuated the increase in the input resistance and the decrease in the threshold current (Figure 8). We noted above (Figure 7) that the increase in the heterogeneity of the ventricular syncytium in the diabetic rats occurs due to a decrease in the density of the* nexus*, which causes an increase in the input resistance values and a decrease in the intracellular thresholds. These changes are supported by the decreased expression of connexin 43, and, therefore, the spatial redistribution of the* nexus* occurs [31, 32]. In the papillary muscles of the diabetic rats with RDP, lower input resistance values and higher current thresholds were found. These magnitudes were similar to those obtained in the control rats. The latter indicates that, in the papillary muscles of the diabetic rats with RDP, there are areas of tissue with a greater density of* nexus*. This observed phenomenon can be explained if we consider that the alterations that occur in the diabetic rats may also occur in dilated cardiomyopathy in the mouse. In this model of dilated cardiomyopathy [26], it was reported that oral supplementation of the mice with an extract of polyphenols contained in the apple increased the expression of connexin 43; consequently, there was an increase in the density and interconnection of the* nexus* in the cardiac myocytes.

Finally, the decrease in the amplitudes of the heterogeneities of the ventricular syncytium and the decrease in the absolute refractory period are two factors that favour the disappearance of the critical propagation of action potentials. Therefore, the heart becomes less vulnerable to arrhythmias. We conclude that apple peel has protective effects on diabetic cardiomyopathy.

## 5. Conclusions

1. The ventricular syncytium of the control rats is heterogeneous.
2. In diabetic cardiomyopathy, the organization of the ventricular syncytium muscle proceeds with functional modifications that are evaluated in terms of input resistance and threshold current.
3. The propagation of premature responses is associated with conduction blocks.
4. The hearts of rats with induced diabetes are more vulnerable to reentrant arrhythmias.
5. The apple peel achieves its protective action by reducing the ARP and attenuating the modifications that the ventricular syncytium suffers during diabetes.

## Figures and Tables

**Table 1:** Analysis of the composition of the lyophilized apple peel (RD).

Component	%
Moisture	81.5
Ash	1.45
Crude protein	0.02
Crude fat	0.35
Carbohydrates	15
Crude fibre	1.59

**Table 2:** Body weight and biochemical parameters of the rats after 90 days of treatment.

	Weight (g)	Glucose (mg/dL)	Insulin (*μ*UI/mL)	HbA_1C_ (%)
Control (*n* = 11)	501.00 ± 13.22	196.50 ± 10.33	0.3455 ± 0.01575	4.282 ± 0.3009
D (*n* = 11)	363.20 ± 29.80^*∗*^	487.10 ± 23.98^*∗*^	0.1545 ± 0.03123^*∗*^	6.009 ± 0.1984^*∗*^
D + RDP (*n* = 8)	366.40 ± 10.67^*∗*^	511.50 ± 58.35^*∗*^	0.1500 ± 0.03727^*∗*^	6.000 ± 0.1753^*∗*^

Mean ± SEM; ^*∗*^ *p* < 0.05, control versus diabetes.

## Abbreviations

APD: Action potential duration
GAE: Gallic acid equivalents
STZ: Streptozotocin
AP: Action potential
ARP: Absolute refractory period
RDP: Red Delicious apple peel.
